# Relation between preoperative aerobic fitness estimated by steep ramp test performance and postoperative morbidity in colorectal cancer surgery: prospective observational study

**DOI:** 10.1093/bjs/znab292

**Published:** 2021-09-18

**Authors:** A. C. M. Cuijpers, A. F. J. M. Heldens, M. J. L. Bours, N. L. U. van Meeteren, L. P. S. Stassen, T. Lubbers, B. C. Bongers

**Affiliations:** Department of Surgery, Maastricht University Medical Centre, Maastricht, the Netherlands; Department of Surgery, School for Oncology and Developmental Biology (GROW), Faculty of Health, Medicine and Life Sciences, Maastricht University, Maastricht, the Netherlands; Department of Physical Therapy, Maastricht University Medical Centre, Maastricht, the Netherlands; Department of Epidemiology, School for Oncology and Developmental Biology (GROW), Faculty of Health, Medicine and Life Sciences, Maastricht University, Maastricht, the Netherlands; Top Sector Life Sciences and Health (Health∼Holland), the Hague, the Netherlands; Department of Anaesthesiology, Erasmus Medical Centre, Rotterdam, the Netherlands; Department of Surgery, Maastricht University Medical Centre, Maastricht, the Netherlands; Department of Surgery, School of Nutrition and Translational Research in Metabolism (NUTRIM), Faculty of Health, Medicine and Life Sciences, Maastricht University, Maastricht, the Netherlands; Department of Surgery, Maastricht University Medical Centre, Maastricht, the Netherlands; Department of Surgery, School for Oncology and Developmental Biology (GROW), Faculty of Health, Medicine and Life Sciences, Maastricht University, Maastricht, the Netherlands; Department of Nutrition and Movement Sciences, School of Nutrition and Translational Research in Metabolism (NUTRIM), Faculty of Health, Medicine and Life Sciences, Maastricht University, Maastricht, the Netherlands; Department of Epidemiology, Care and Public Health Research Institute (CAPHRI), Faculty of Health, Medicine and Life Sciences, Maastricht University, Maastricht, the Netherlands

## Abstract

Steep ramp test (SRT) performance provides an estimation of preoperative aerobic fitness that is associated with postoperative outcomes. Patients with a better SRT-estimated aerobic fitness are less likely to develop postoperative complications and more likely to experience a shorter time to recovery. The SRT might be a useful and clinically accessible tool in preoperative risk assessment to identify patients at risk of postoperative morbidity and who might benefit from preoperative exercise interventions.

## Introduction

Surgical resection is the mainstay of curative treatment for colorectal cancer. Despite extensive preoperative risk assessment, the risk of postoperative complications remains high^1^. Improvement of preoperative physical fitness, especially aerobic fitness, is now considered to be a potentially modifiable risk factor. Low preoperative aerobic fitness, assessed by cardiopulmonary exercise testing (CPET), is associated with an increased risk of postoperative complications after abdominal surgery^2^^,^^3^, and might indicate a decreased ability to cope with surgical stress. Improving aerobic fitness before surgery in high-risk patients, referred to as exercise prehabilitation, potentially lowers the risk of postoperative morbidity by enabling patients to better withstand perioperative stressors^4–6^.

To offer timely exercise prehabilitation, early identification of patients at risk of postoperative morbidity based on low aerobic fitness is needed. Because CPET is not widely available and is relatively expensive in terms of equipment and personnel, an easy-to-perform preoperative aerobic fitness assessment to evaluate postoperative morbidity risk is attractive. The steep ramp test (SRT) is a potential candidate for this purpose. The SRT is a short-time maximal cycle ergometer test that correlates highly with aerobic fitness assessed by CPET in adult cancer survivors^7^^,^^8^. However, associations between SRT performance and postoperative morbidity in patients with colorectal cancer have not yet been established.

This study aimed to evaluate the associations between SRT-estimated preoperative aerobic fitness and postoperative complications, time to recovery of physical functioning, and duration of hospital stay in patients scheduled for elective colorectal cancer surgery.

## Methods

A complete description of the study methodology is available in *Appendix S1*. In brief, patients diagnosed with colorectal cancer and scheduled for elective resection were referred for a preoperative physical fitness assessment as part of usual care. Assessments to estimate preoperative aerobic fitness included a modified SRT^7^ (work rate increments of 10 W/10 s), a 2-min walk test (2MWT)^9^, and the Duke Activity Status Index (DASI)^10^. Before undertaking statistical analyses, potential confounders were identified including age, sex, BMI, co-morbidities, neoadjuvant treatment, tumour location, and surgical approach. Postoperative outcome measures were occurrence of complications, graded by the Clavien–Dindo classification^11^, time to recovery of physical functioning (in days), assessed by the modified Iowa Level of Assistance Scale (mILAS) (a mILAS score of 0 reflects recovery of physical functioning)^12^, and duration of hospital stay.

## Results

**Fig. 1:**
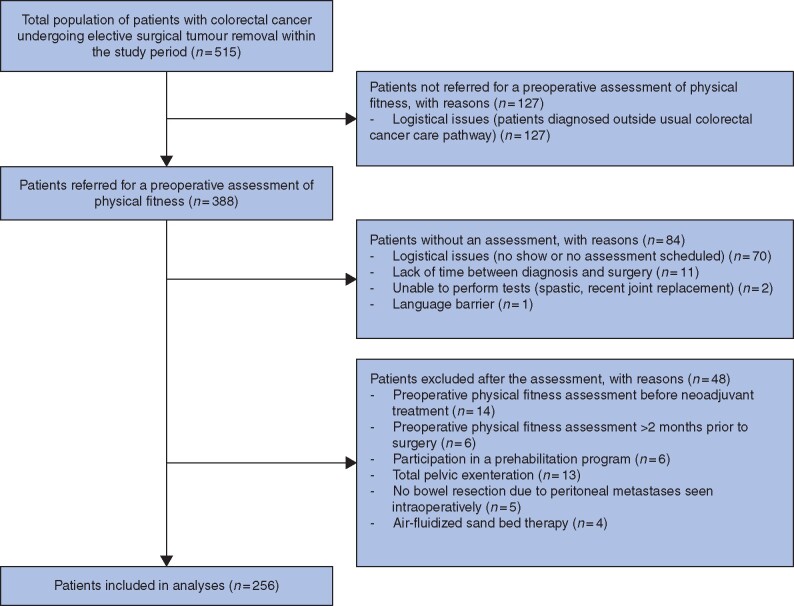
Study flow chart

Of 304 consecutive patients who had a preoperative physical fitness assessment between January 2016 and March 2020, 256 met the inclusion criteria and were included in the analysis (*Fig. 1*). Baseline characteristics and postoperative outcomes are shown in *Table 1*. *Table S1* provides a comparison of baseline characteristics and postoperative outcomes of included and excluded patients.

**Table 1:** Baseline characteristics and postoperative recovery

		Complications			*P*
	Overall (*n* = 256)	Yes (*n* = 107)	No (*n* = 149)		
Age (years)*	69.4(10.0)	69.9(9.7)	69.0(10.1)		0.446◊
**Sex**					0.102◊◊◊
M	145 (56.6)	67 (62.6)	78 (52.3)		
F	111 (43.4)	40 (37.4)	71 (47.7)		
BMI (kg/m^2^)*	26.9(5.0)	27.3(5.0)	26.6(5.0)		0.292◊
Preoperative haemoglobin (g/dl)*‡	12.8(1.9)	12.8(1.9)	12.8(2.0)		0.911◊
**Smoker**§	33 (13.0)	13 (12.1)	20 (13.4)		0.792◊◊◊
**ASA fitness grade**					0.005◊◊◊
I	23 (9.0)	5 (4.7)	18 (12.1)		
II	164 (64.1)	63 (58.9)	101 (67.8)		
III	69 (27.0)	39 (36.4)	30 (20.1)		
IV	0 (0)				
**Charlson Co-morbidity Index score**†	3 (3–5)	4 (3–6)	3 (2–5)		0.040◊◊
**Neoadjuvant radiotherapy**	56 (21.9)	30 (28.0)	26 (17.4)		0.043◊◊◊
**Neoadjuvant chemotherapy**	49 (19.1)	29 (27.1)	20 (13.4)		0.006◊◊◊
**Tumour location**					0.008◊◊◊
Colon	165 (64.5)	59 (55.1)	106 (71.1)		
Rectum	91 (35.5)	48 (44.9)	43 (28.9)		
**Surgical approach**					0.083◊◊◊
Laparoscopy/robot (assisted)	230 (89.8)	92 (86.0)	138 (92.6)		
Laparotomy	26 (10.2)	15 (14.0)	11 (7.4)		
**Surgical procedure**					0.002◊◊◊
Right hemicolectomy	95 (37.1)	36 (33.6)	59 (39.6)		
Transverse hemicolectomy	4 (1.6)	2 (1.9)	2 (1.3)		
Left hemicolectomy	15 (5.9)	7 (6.5)	8 (5.4)		
Sigmoid resection	42 (16.4)	7 (6.5)	35 (23.5)		
Subtotal colectomy	5 (2.0)	5 (4.8)	0 (0)		
Low anterior resection‡‡	71 (27.7)	37 (34.6)	34 (22.8)		
Abdominoperineal resection	16 (6.3)	8 (7.5)	8 (5.4)		
Proctocolectomy	1 (0.4)	1 (0.9)	0 (0)		
Other§§	7 (2.7)	4 (3.7)	3 (2.0)		
**Preoperative physical fitness**					
SRT WR_peak_ (W)*¶	173.23(58.74)	165.28(59.71)	178.39(57.78)		0.135◊
SRT WR_peak_ (W/kg)*¶	2.26(0.76)	2.10(0.74)	2.37(0.75)		0.020◊
2MWT (m)†#	165 (138–161.5)	165 (130–180)	165 (142–195)		0.043◊◊
DASI (MET)†,**	7.99 (6.36–8.79)	7.99 (5.62–8.97)	8.23 (6.93–9.89)		0.031◊◊
**Postoperative recovery**					
Time to mILAS = 0 (days)†,††	4 (3–7)	8 (5–15)	3 (2–4)		<0.001◊◊
Duration of hospital stay (days)†	6 (4–11)	13 (7–22)	4 (3–5)		<0.001◊◊
Readmission (yes)	31 (12.1)	29 (27.1)	2 (1.3)		<0.001◊◊◊

Values in parentheses are percentages unless indicated otherwise; values are *mean(s.d.) and †median (i.q.r.). Data shown for ‡241 (complications yes: 104, no: 137), §253 (complications yes: 105, no: 148), ¶188 (complications yes: 74, no: 114), #250 (complications yes: 103, no: 147), **251 (complications yes: 105, no: 146), ††254 (complications yes: 105, no: 149). ‡‡By total or partial mesenteric excision. §§Surgical removal of bowel parts simultaneously. SRT, steep ramp test; WR_peak_, peak work rate; 2MWT, 2-min walk test; DASI, Duke Activity Status Index; MET, metabolic equivalent of task; mILAS, modified Iowa Level of Assistance Scale. ◊Independent samples T test, ##◊◊Mann-Withney U test, ◊◊◊χ^2^ test.

### Preoperative steep ramp test performance and postoperative complications

Postoperative complications (Clavien–Dindo grade I or higher) occurred in 107 patients (41.7 per cent). *Table S2* provides an overview of all complications. Patients with postoperative complications had lower preoperative SRT performance (mean(s.d.) 2.10(0.74) *versus* 2.37(0.75) W/kg in patients without complications; *P* = 0.020) (*Table 1*). Hierarchical binary logistic regression analysis showed that a lower SRT performance was associated with postoperative complications (odds ratio (OR) 0.50, 95 per cent c.i. 0.26 to 0.96; *P* = 0.038) after adjustment for prespecified confounders (*Table 2*). Preoperative 2MWT and DASI scores were lower in patients with postoperative complications (*P* = 0.043 and *P* = 0.031 respectively). Comparable to SRT performance, lower 2MWT and DASI scores were associated with a higher risk of postoperative complications on univariable analysis, but not in the multivariable models (OR 0.99, 0.98 to 1.00, *P* = 0.135; OR 0.85, 0.71 to 1.02, *P* = 0.080).

**Table 2:** Logistic regression analysis for postoperative complications, time to recovery of physical functioning, and duration of hospital stay

	Postoperative complications (Clavien–Dindo ≥ I)		Time to mILAS = 0		Duration of hospital stay	
	Odds ratio	*P*	Odds ratio	*P*	Odds ratio	*P*
**SRT WR_peak_ (W/kg)**						
Model 1*	0.62 (0.41, 0.93)	0.022	0.58 (0.38, 0.89)	0.012	0.73 (0.49, 1.09)	0.124
Model 2†	0.46 (0.25, 0.87)	0.018	0.38 (0.20, 0.73)	0.004	0.50 (0.27, 0.94)	0.031
Model 3‡	0.50 (0.26, 0.96)	0.038	0.36 (0.18, 0.71)	0.003	0.55 (0.29, 1.05)	0.070
**2MWT (m)**						
Model 1*	0.99 (0.99, 1.00)	0.038	0.99 (0.98, 1.00)	0.001	0.99 (0.98, 1.00)	0.005
Model 2†	0.99 (0.98, 1.00)	0.060	0.99 (0.98, 1.00)	0.002	0.99 (0.99, 1.00)	0.03
Model 3‡	0.99 (0.98, 1.00)	0.135	0.99 (0.98, 1.00)	0.006	0.99 (0.98, 1.00)	0.011
**DASI (MET)**						
Model 1*	0.85 (0.74, 0.98)	0.021	0.79 (0.69, 0.92)	0.002	0.85 (0.74, 0.98)	0.027
Model 2†	0.82 (0.69, 0.98)	0.026	0.79 (0.66, 0.94)	0.008	0.82 (0.89, 0.98)	0.026
Model 3‡	0.85 (0.71, 1.02)	0.080	0.82 (0.68, 0.98)	0.031	0.86 (0.72, 1.04)	0.177

Values in parentheses are 95 per cent confidence intervals. mILAS, modified Iowa Level of Assistance Scale; SRT, steep ramp test; WR_peak_, peak work rate; 2MWT: 2-min walk test. DASI, Duke Activity Status Index; MET, metabolic equivalent of task. *Model 1: unadjusted univariable analysis. †Model 2: adjusted for age, sex, BMI, and Charlson Co-morbidity Index. ‡Model 3: fully adjusted multivariable analysis, adjusted for age, sex, BMI, Charlson Co-morbidity Index, surgical procedure (laparoscopic/robotic *versus* laparotomy), tumour location (colon *versus* rectum), neoadjuvant chemotherapy, and neoadjuvant radiotherapy.

### Preoperative steep ramp test performance, time to recovery of physical functioning, and hospital stay

Median time to mILAS = 0 and median duration of hospital stay were 4 and 6 days respectively. Time to mILAS = 0 and length of stay were dichotomized as no more than 4 or at least 5 days and no more than 6 or at least 7 days respectively. Better preoperative SRT performance was associated with shorter time to mILAS = 0 in the fully adjusted analysis (OR 0.36, 95 per cent c.i. 0.18 to 0.71; *P* = 0.003). Better preoperative SRT performance was also associated with shorter hospital stay when adjusted for age, sex, BMI, and co-morbidities (OR 0.50, 0.27 to 0.94; *P* = 0.031) in univariable analysis, but not in the fully adjusted model (OR 0.55, 0.29 to 1.05; *P* = 0.070). Comparable associations were seen for 2MWT and DASI scores for both mILAS = 0 and duration of hospital stay (*Table 2*).

## Discussion

In this study, SRT-estimated preoperative aerobic fitness was inversely associated with postoperative complications. Patients with a higher SRT performance were less likely to develop postoperative complications, with the lowest OR found in the fully confounder-adjusted model. Associations between 2MWT and DASI scores and postoperative outcomes were assessed comparatively; these variables were associated with the occurrence of postoperative complications in univariable analysis. Despite loss of statistical significance in the adjusted models, the similar direction of observed associations strengthens the likelihood of an inverse association between preoperative estimated aerobic fitness and risk of postoperative complications. Additionally, SRT performance was inversely associated with the time to recovery of physical functioning, which also supports the relevance of preoperative aerobic fitness in relation to postoperative morbidity.

The present results highlight the value of preoperative aerobic fitness assessment as a risk estimator for postoperative morbidity in abdominal cancer surgery, supporting its incorporation into clinical practice. The relationship between field exercise tests and postoperative morbidity has been evaluated previously; however, evidence of their usefulness in preoperative risk assessment is based on small sample sizes^13–16^. CPET is an established identifier of patients at risk of postoperative morbidity^2^^,^^3^, but is often not feasible in all institutions. Compared with CPET, the SRT is a short and easily accessible maximal exercise test without respiratory gas analysis, equally useful for prescribing training load and measuring training progress^8^. Where preoperative aerobic fitness is increasingly recognized as a risk estimator and target for preoperative intervention in terms of prehabilitation, the SRT appears to be a promising tool for clinical implementation. Research to validate preoperative SRT performance compared with preoperative CPET in patients with colorectal cancer scheduled for elective resection is ongoing.

Low preoperative aerobic fitness might be substantial in patients with colorectal cancer, with the majority of this population aged over 60 years^17^. Therefore, future research should assess how to incorporate aerobic fitness into preoperative risk assessments. Every patient is characterized by a profile of (un)modifiable risk factors. It is unlikely that low preoperative aerobic fitness alone sufficiently predicts adverse postoperative outcomes. Preoperative haemoglobin levels, nutritional status, fatigue, psychosocial well-being, frailty, and factors related to systemic inflammation and sarcopenia might also be considered for inclusion in preoperative risk assessment and multimodal prehabilitation programmes to improve preoperative risk prediction and subsequently postoperative outcome^6^^,^^18^. Combining aerobic fitness with other (un)modifiable risk factors in prediction models for postoperative morbidity might further improve identification of high-risk patients and guide patient-tailored prehabilitation.

Along with predicting complication risk, identifying a patient’s resilience to potential complications might be another valuable feature of preoperative aerobic fitness. Fitter patients appear to cope better with the impact of complications, resulting in a faster recovery^5^^,^^15^^,^^19^^,^^20^. Future research to predict a patient’s resilience to complications might be as important as the prediction of complications itself.

Limitations of this study were a difference in ASA classification between included and excluded patients (*Table S1*), and potential selection bias. Selection bias was expected to be limited because the preoperative physical fitness assessment was part of usual care.

## Supplementary Material

znab292_Supplementary_Data
