# Supplementary material for: Relation between preoperative aerobic fitness estimated by steep ramp test performance and postoperative morbidity in colorectal cancer surgery: prospective observational study
Source: Br J Surg. 2021 Sep 18;109(2):155–9. doi: 10.1093/bjs/znab292 (PMC10364754; doi:10.1093/bjs/znab292)
Supplement: znab292_Supplementary_Data [file znab292_supplementary_data.zip › Appendix S1.docx]

**Appendix S1**

**Methods**

This was a single centre prospective observational cohort study conducted at Maastricht University Medical Centre+, Maastricht, The Netherlands and reported consistent with the STrengthening the Reporting of OBservational studies in Epidemiology (STROBE) statement.^1, 2^ Ethical approval was obtained by the Medical Ethical Committee of Maastricht University Medical Centre/Maastricht University (15-4-234).

*Study population*

Patients diagnosed with colorectal cancer (CRC) and scheduled for elective resection were referred to the outpatient physical therapy department by the colorectal nurse specialist or the surgeon for a preoperative physical fitness assessment as part of usual care. All consecutive patients who were aged ≥ 18 years and agreed to the use of their usual care data for research purposes by providing informed consent were eligible and were prospectively recorded in a database. Patient characteristics at time of surgery (date of birth, sex, body mass index (BMI), comorbidities, and American Society of Anaesthesiologists (ASA) physical status classification), tumour characteristics, treatment details, and postoperative outcomes (e.g. complications, reinterventions, intensive care admissions, readmissions, and death) are prospectively recorded for national registration purposes. Data from eligible patients were extracted from this database and checked for accuracy and completeness using the electronic patient files. Charlson comorbidity index (CCI) was used to quantify comorbidities.^3^ Data was collected between January 2016 and March 2020. The number of referred patients during the study period determined the sample size. Patients were excluded in case of no bowel resection due to peritoneal metastases, total pelvic exenteration, a preoperative assessment of physical fitness before neoadjuvant chemoradiotherapy or >2 months prior to surgery, participation in a prehabilitation program, or postoperative air-fluidized sand bed therapy. To account for potential bias, baseline characteristics and postoperative outcome measures of the patients not included in the analysis were compared to the included patients.

*Preoperative physical fitness assessment*

As part of usual care, a preoperative assessment of physical fitness was conducted by a hospital physical therapist with expertise in the colorectal surgical field. During the assessment, aerobic fitness was estimated by the steep ramp test (SRT) on a calibrated cycle ergometer (Lode Corival Rehab, Lode BV, Groningen, the Netherlands). Seat height was set at comfortable height for each individual. To make the SRT suitable for our surgical population, the original protocol (work rate increments of 25 W each 10 s) was modified.^4^ After two minutes of unloaded cycling, the work rate was increased by constant increments of 10 W each 10 s in a ramp like manner of 1 W/s.^5^ Throughout the test, patients were instructed to keep a pedalling frequency between 70 and 80 revolutions/min. The protocol continued until the patient’s pedalling frequency fell definitely below 60 revolutions/min, despite strong verbal encouragement. The attained peak work rate (WR_peak_) adjusted for body mass, as an indicator of aerobic fitness, was the primary outcome of the SRT.

Additionally, functional walking capacity was assessed using the two-minute walk test (2MWT), while perceived functional capacity was evaluated using the Duke activity status index (DASI). The 2-MWT is a submaximal exercise test recording the distance walked over a length of 15 meters in two minutes.^6^ The DASI is a 12-item questionnaire assessing self-recorded functional capacity based on the patient’s activities of daily living.^7^ Both measures show a moderate-to-good association with aerobic fitness (oxygen consumption at peak exercise (VO_2peak_)) in different patient populations, including patients undergoing elective abdominal surgery.^8-10^

*Postoperative care*

Postoperative care was similar for all patients and in accordance to the enhanced recovery after surgery (ERAS) protocol.^11^ All patients received postoperative physical therapy starting at postoperative day 1 which consisted of transfers, walking, stair climbing (when necessary for independent functioning at home) and exercising airway clearing (when necessary). Additionally, exercises for recovery of muscle function and aerobic fitness were performed.

*Outcome variables*

The primary outcome was the presence of postoperative complications, which were recorded using the Clavien-Dindo classification.^12^ Postoperative complications were defined as a Clavien-Dindo grade ≥I (CD ≥I). Secondary outcomes were time to recovery of physical functioning and length of hospital stay (LOS). Recovery of physical functioning was monitored daily by the hospital physical therapist using the modified Iowa level of assistance scale (mILAS).^13^ This scale assesses the ability of patients to perform five daily activities (supine-to-sit, sit-to-supine, sit-to-stand, walking, and stair climbing) and records the level of assistance needed for each activity. Time to recovery of physical functioning was defined as the time in days between the day of surgery and the day a patient reached a mILAS score of 0 (mILAS=0). LOS was defined as the number of days from the day of surgery until hospital discharge.

*Statistical analysis*

Statistical analysis was performed using IBM SPSS Statistics for Windows, version 26 (IBM Corp., Armonk, N.Y., USA). For all continuous variables, normality was tested using histograms, Q-Q plots, and Kolmogorov-Smirnov tests. Values were displayed as mean ± standard deviation (SD) or as median and interquartile range [IQR], as appropriate. Independent samples t-tests or Mann-Whitney *U* tests were used for continuous variables, according to normality. χ2 and Fisher exact tests were used for categorical values. Hierarchical binary logistic regression analysis was used to assess the association between preoperative estimated aerobic fitness and postoperative morbidity, adjusted for confounding. Results are presented as odds ratios (OR) with 95% confidence intervals (CI). Prior to multivariable logistic regression analysis, eight preoperative variables were identified as potential confounders for the relation between preoperative estimated aerobic fitness and postoperative morbidity. Confounders were identified based on existing literature and expert opinion and were hierarchically added to the logistic regression model. Potential confounders included age, sex, BMI, neoadjuvant chemotherapy or radiotherapy, tumour location (colon or rectum), surgical approach (laparoscopy/robot (assisted) or laparotomy), and CCI. SRT performance (WR_peak_ in W/kg), 2MWT (m), and DASI scores (MET) were included in the analysis as continuous variables. Data on postoperative outcomes were dichotomized. Complications were classified as no complications or CD ≥I. Time to recovery of physical functioning (mILAS=0, in days) and length of hospital stay (in days) were dichotomized based on their respective median value in the current study population. Two-tailed p-values <0.05 were considered statistically significant.

**References**

1. Vandenbroucke JP, von Elm E, Altman DG, Gøtzsche PC, Mulrow CD, Pocock SJ, et al. Strengthening the Reporting of Observational Studies in Epidemiology (STROBE): Explanation and Elaboration. *PLOS Medicine*. 2007; **4**: e297.

2. von Elm E, Altman DG, Egger M, Pocock SJ, Gøtzsche PC, Vandenbroucke JP, et al. The Strengthening the Reporting of Observational Studies in Epidemiology (STROBE) Statement: Guidelines for Reporting Observational Studies. *PLOS Medicine*. 2007; **4**: e296.

3. Charlson ME, Pompei P, Ales KL, MacKenzie CR. A new method of classifying prognostic comorbidity in longitudinal studies: development and validation. *Journal of chronic diseases*. 1987; **40**: 373-83.

4. Meyer K, Samek L, Schwaibold M, Westbrook S, Hajric R, Lehmann M, et al. Physical responses to different modes of interval exercise in patients with chronic heart failure--application to exercise training. *Eur Heart J*. 1996; **17**: 1040-7.

5. Van Beijsterveld CA, Bongers BC, Den Dulk M, Van Kuijk SMJ, Dejong KCH, Van Meeteren NLU. The association between preoperative physical functioning and short-term postoperative outcomes: a cohort study of patients undergoing elective hepatic resection. *HPB (Oxford)*. 2019; **21**: 1362-70.

6. Butland RJ, Pang J, Gross ER, Woodcock AA, Geddes DM. Two-, six-, and 12-minute walking tests in respiratory disease. *British Medical Journal (Clinical research ed)*. 1982; **284**: 1607.

7. Hlatky MA, Boineau RE, Higginbotham MB, Lee KL, Mark DB, Califf RM, et al. A brief self-administered questionnaire to determine functional capacity (the Duke Activity Status Index). *The American journal of cardiology*. 1989; **64**: 651-4.

8. Negm MF, Abdalla ME, Almahdy MA. Study of 2-min walk test and 15-step exercise oximetry test in the assessment of exercise tolerance in Egyptian patients with chronic obstructive pulmonary disease. *Egyptian Journal of Chest Diseases and Tuberculosis*. 2012; **61**: 291-6.

9. Ricci PA, Cabiddu R, Jürgensen SP, André LD, Oliveira CR, Di Thommazo-Luporini L, et al. Validation of the two-minute step test in obese with comorbibities and morbidly obese patients. *Braz J Med Biol Res*. 2019; **52**: e8402.

10. Struthers R, Erasmus P, Holmes K, Warman P, Collingwood A, Sneyd JR. Assessing fitness for surgery: a comparison of questionnaire, incremental shuttle walk, and cardiopulmonary exercise testing in general surgical patients. *British journal of anaesthesia*. 2008; **101**: 774-80.

11. Gustafsson UO, Scott MJ, Hubner M, Nygren J, Demartines N, Francis N, et al. Guidelines for Perioperative Care in Elective Colorectal Surgery: Enhanced Recovery After Surgery (ERAS(®)) Society Recommendations: 2018. *World J Surg*. 2019; **43**: 659-95.

12. Dindo D, Demartines N, Clavien PA. Classification of surgical complications: a new proposal with evaluation in a cohort of 6336 patients and results of a survey. *Annals of surgery*. 2004; **240**: 205-13.

13. Shields RK, Enloe LJ, Evans RE, Smith KB, Steckel SD. Reliability, Validity, and Responsiveness of Functional Tests in Patients With Total Joint Replacement. *Physical Therapy*. 1995; **75**: 169-76.
