# Supplementary material for: Relation between preoperative aerobic fitness estimated by steep ramp test performance and postoperative morbidity in colorectal cancer surgery: prospective observational study
Source: Br J Surg. 2021 Sep 18;109(2):155–9. doi: 10.1093/bjs/znab292 (PMC10364754; doi:10.1093/bjs/znab292)
Supplement: znab292_Supplementary_Data [file znab292_supplementary_data.zip › Table S1 - Baseline characteristics excluded patients.docx]

| **Table S1: Baseline characteristics included versus excluded cases** | | | |
| --- | --- | --- | --- |
|  | **Included cases (n=256)** | **Not included cases (n=259)** | ***p* value** |
| **Age (years)** | 69.4 ± 10.0 | 68.2 ± 11.4 | 0.231 |
| **Sex^a^** |  |  | 0.560 |
| Male | 145 (56.6%) | 113 (59.8%) |  |
| Female | 111 (43.4%) | 76 (40.2%) |  |
| **BMI (kg/m^2^)** | 26.9 ± 5.0 | 26.7 ± 4.9 | 0.599 |
| **Presence of comorbidities (yes)** | 160 (62.5%) | 162 (62.5%) | 1.000 |
| **ASA classification** |  |  | ***0.013*** |
| I | 23 (9.0%) | 13 (5.0%) |  |
| II | 164 (64.1%) | 146 (56.4%) |  |
| III | 69 (27.0%) | 98 (37.8%) |  |
| IV | 0 | 2 (0.8%) |  |
| **Location tumour** |  |  | 0.381 |
| Colon | 165 (64.5%) | 155 (59.8%) |  |
| Rectum | 91 (35.5%) | 104 (40.2%) |  |
| **Complications (yes)** | 107 (41.7%) | 106 (40.9%) | 0.858 |
| **Reintervention (yes)** | 40 (15.7%) | 42 (16.2%) | 0.904 |
| **ICU admission (yes)** | 16 (6.3%) | 17 (6.6%) | 0.776 |
| **LOS** | 6 [4; 11] | 7 [4; 13] | 0.238 |
| **Readmission (yes)** | 31 (12.1%) | 32 (12.4%) | 0.893 |
| Data displayed as absolute number (%), mean ± SD, or median [IQR].  ^a^: n=189 in the not included cases group.  Abbreviations: ASA: American Society of Anaesthesiologists physical status classification, BMI: body mass index, ICU: intensive care unit, LOS: length of hospital stay. | | | |
