# Supplementary material for: Relation between preoperative aerobic fitness estimated by steep ramp test performance and postoperative morbidity in colorectal cancer surgery: prospective observational study
Source: Br J Surg. 2021 Sep 18;109(2):155–9. doi: 10.1093/bjs/znab292 (PMC10364754; doi:10.1093/bjs/znab292)
Supplement: znab292_Supplementary_Data [file znab292_supplementary_data.zip › Table S2 - Postoperative complications in total population.docx]

| **Table S2: Postoperative complications in total population (n=256)** | | |
| --- | --- | --- |
| **Surgical complications** | 78 | (30.5%) |
| Anastomotic leakage | 21 | (8.2%) |
| Abscess | 22 | (8.6%) |
| Bleeding | 8 | (3.1%) |
| Ileus | 45 | (17.6%) |
| Fascial dehiscence | 4 | (1.6%) |
| Bowel perforation | 1 | (0.4%) |
| Wound infection | 14 | (5.5%) |
| Other | 19 | (7.4%) |
| **Non-surgical complications** | 61 | (23.8%) |
| Pulmonary | 18 | (7.0%) |
| Cardiac | 15 | (5.9%) |
| Thrombotic | 5 | (2.0%) |
| Infectious | 19 | (7.4%) |
| Neurologic | 8 | (3.1%) |
| Other | 20 | (7.8%) |
| **Clavien-Dindo Classification** |  |  |
| I | 21 | (8.2%) |
| II | 42 | (16.4%) |
| IIIa | 9 | (3.5%) |
| IIIb | 19 | (7.4%) |
| IVa | 12 | (4.7%) |
| IVb | 2 | (0.8%) |
| V | 2 | (0.8%) |
| **Comprehensive complication index** | 0.00 | [0.00; 20.92] |
| Comprehensive complication index (patients with complications only)^a^ | 26.22 | [20.92; 42.43] |
| **Re-intervention** | 40 | (15.6%) |
| Radiological | 4 | (1.6%) |
| Endoscopic | 4 | (1.6%) |
| Laparoscopy | 2 | (0.8%) |
| Laparotomy | 24 | (9.4%) |
| Other | 6 | (2.3%) |
| **ICU admission** | 16 | (6.3%) |
| Single organ failure | 10 | (3.9%) |
| Multi organ failure | 4 | (1.6%) |
| Other | 2 | (0.8%) |
| Data displayed as median [IQR] or absolute number (%).  Abbreviations: ICU: intensive care unit.  ^a^: n=107 | | |
